# Cardiorenal Syndrome in a Kidney Transplant Recipient With High-Flow Arteriovenous Fistula and Mycotic Pneumonia

**DOI:** 10.7759/cureus.107627

**Published:** 2026-04-24

**Authors:** Jan Broda, Bartosz Foroncewicz, Eliza Bak, Szymon Piotrowski, Krzysztof Mucha

**Affiliations:** 1 Department of Transplantology, Immunology, Nephrology and Internal Medicine, Medical University of Warsaw, Warsaw, POL

**Keywords:** arterivenous fistula, cardiorenal syndrome (crs), deterioration of kidney graft function, haemodialysis access, kidney transplant recipient, post-transplantation care, pulmonary mycosis

## Abstract

The arteriovenous fistula (AVF) is a preferred access for haemodialysis (HD) in patients with end-stage renal disease. However, its haemodynamic effect on the heart is not negligible; it may cause or exacerbate heart failure and pulmonary hypertension, especially when the flow is excessively high. After the transplantation, a decrease in cardiac output leads to reduced kidney graft perfusion, which can result in the development of cardiorenal syndrome. The guidelines for managing AVF after transplantation are still not well defined, so care and surveillance are based on the experience of transplantation centres. We present a case of a kidney transplant recipient with deterioration of graft function and sepsis. He received a kidney graft in 2005, and since then, he has had a functioning enlarged AVF, which has not been used since the transplantation. At the time of admission, the flow through the fistula exceeded 7000 mL/min. Blood culture results suggested *Staphylococcus haemolyticus *as the causative pathogen. The patient was treated with a prolonged course of meropenem and vancomycin based on the antibiogram. Due to radiological findings suggestive of mycotic pneumonia, voriconazole was added. Because of cardiac failure, he was disqualified from surgical treatment of the mycotic pneumonia. He required repeated HD and ultrafiltration for a month using a non-tunnelled catheter. To treat cardiac insufficiency and improve graft function, the fistula was surgically removed. After the surgery, the patient no longer required HD, and his serum creatinine concentration decreased. This case demonstrates that graft function might be restored even after prolonged HD. Therefore, the decision whether to discontinue immunosuppressive therapy to treat the infection must be made carefully. Ligation of a high-flow AVF may contribute to the treatment of cardiac insufficiency, thereby improving graft function. AVF should be monitored regularly to prevent its adverse effects on both cardiac and graft function, which could complicate the management of emerging conditions, as in this case, mycotic pneumonia.

## Introduction

Despite the recognised adverse effects of the arteriovenous fistula (AVF) on cardiac function, it remains the gold standard for vascular access in haemodialysis (HD) [1]. However, high-flow AVF causes diversion of blood from arteries to the lower-resistance venous circulation, increasing venous return to the right atrium and right ventricle output. It contributes to the development of pulmonary hypertension and heart failure (HF) in kidney transplant recipients (KTRs). Moreover, it is suggested that tricuspid regurgitation caused by the remodelled right ventricle leads to increased pressure in the inferior vena cava and iliac veins, contributing to a decrease in glomerular filtration rate of the graft, although there is not enough evidence confirming that hypothesis [2]. The interdependence between heart and kidney function and the disruption of this interaction is widely described and classified in the literature as cardiorenal syndrome [3]. Deterioration of one organ can cause dysfunction of the other and progression of insufficiency in both. Given that cardiovascular diseases are still the leading cause of death in KTRs, with an incidence five times higher than in the general population [4], attention to the management of the AVF must be given. However, there is no consensus on how to manage AVF after transplantation [5,6]. In 2025, the Vascular Access Society, together with the European Kidney Transplant Association, proposed an algorithm for managing uncomplicated AVF, also emphasising the need for prospective studies to establish guidelines and a surveillance protocol for AVF after transplantation [7]. This case report aims to illustrate how high-flow AVF can contribute to cardiorenal dysfunction and complicate the management of concomitant severe infection in a KTR. We also want to emphasise the importance of AVF management after transplantation.

## Case presentation

A 54-year-old KTR was admitted to the transplantation department in July 2024 due to pneumonia difficult to treat in the outpatient clinic and deterioration of graft function (serum creatinine (SCr) 3.39 mg/dL, estimated glomerular filtration rate (eGFR) 19 mL/min/1.73 m², and hyperkalaemia). He received a kidney transplant in August 2005 because of end-stage renal disease of unknown aetiology (glomerulonephritis was suspected). His maintenance immunosuppression included prednisone, tacrolimus, and mycophenolate mofetil.

In 2006, a graft biopsy was performed because of proteinuria and microhaematuria, revealing membranous nephropathy and focal segmental glomerulosclerosis. In 2017, a second biopsy was carried out due to deterioration in graft function and proteinuria. Thrombotic microangiopathy with a complex aetiology (polymorphism in the methylenetetrahydrofolate reductase gene), cyclosporine-dependent, was diagnosed. Since 2022, stable graft dysfunction has been observed, with SCr fluctuating between 2.5 and 3 mg/dL (eGFR from 35 to 30 mL/min/1.73 m²). In June 2024, he experienced an episode of diffuse alveolar haemorrhage, with no signs of infection. Based on computed tomography (CT), vasculitis was diagnosed, although antineutrophil cytoplasmic antibodies (ANCA) and antinuclear antibodies (ANA) were negative. He was treated with methylprednisolone (three doses of 500 mg intravenously) followed by prednisone (1 mg per kg body weight). He reduced the therapy on his own due to leg oedema and severe labial herpes.

**Figure 1:**
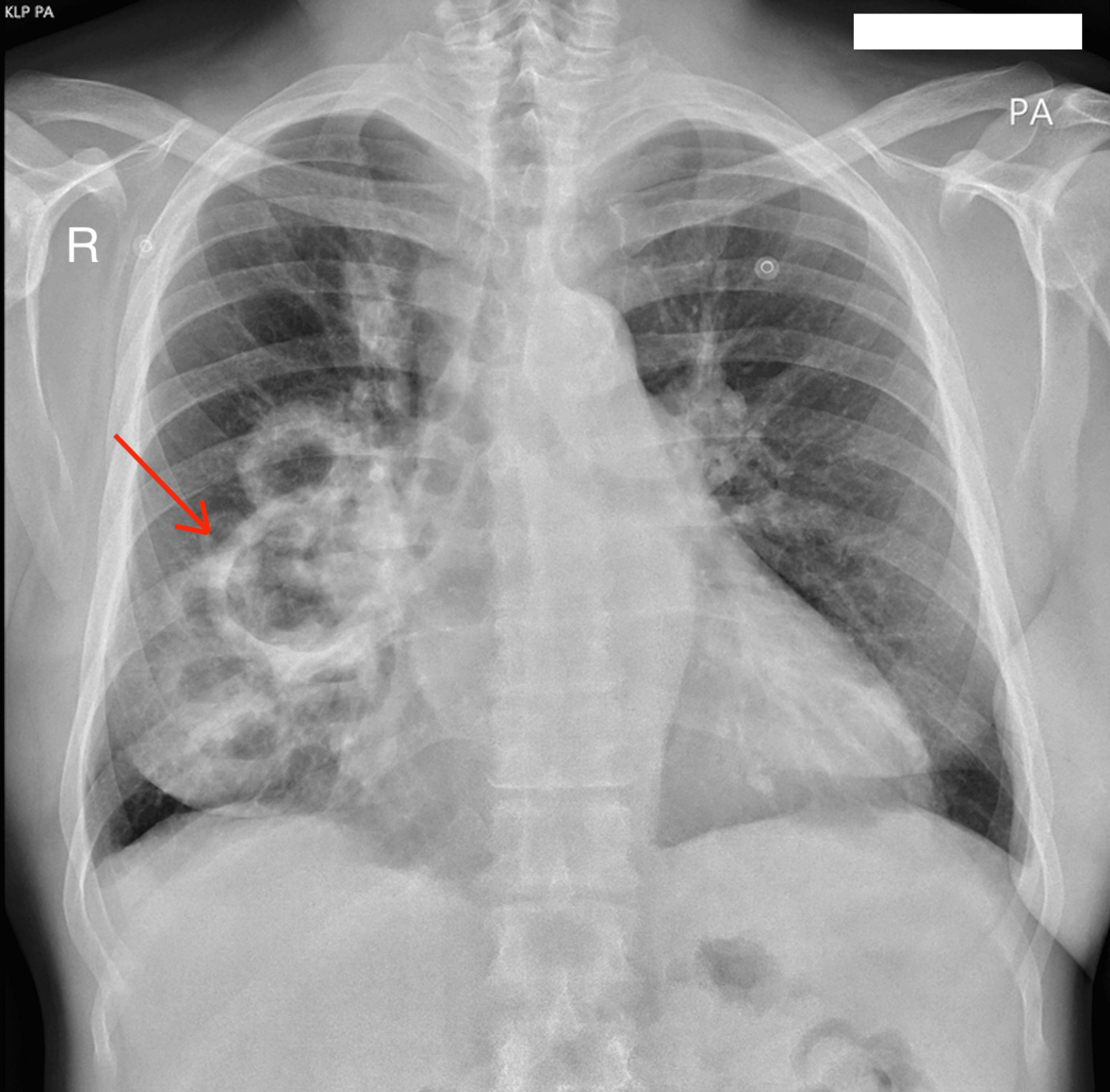
Chest X-ray on admission with visible lesion in right lung (red arrow).

**Figure 2:**
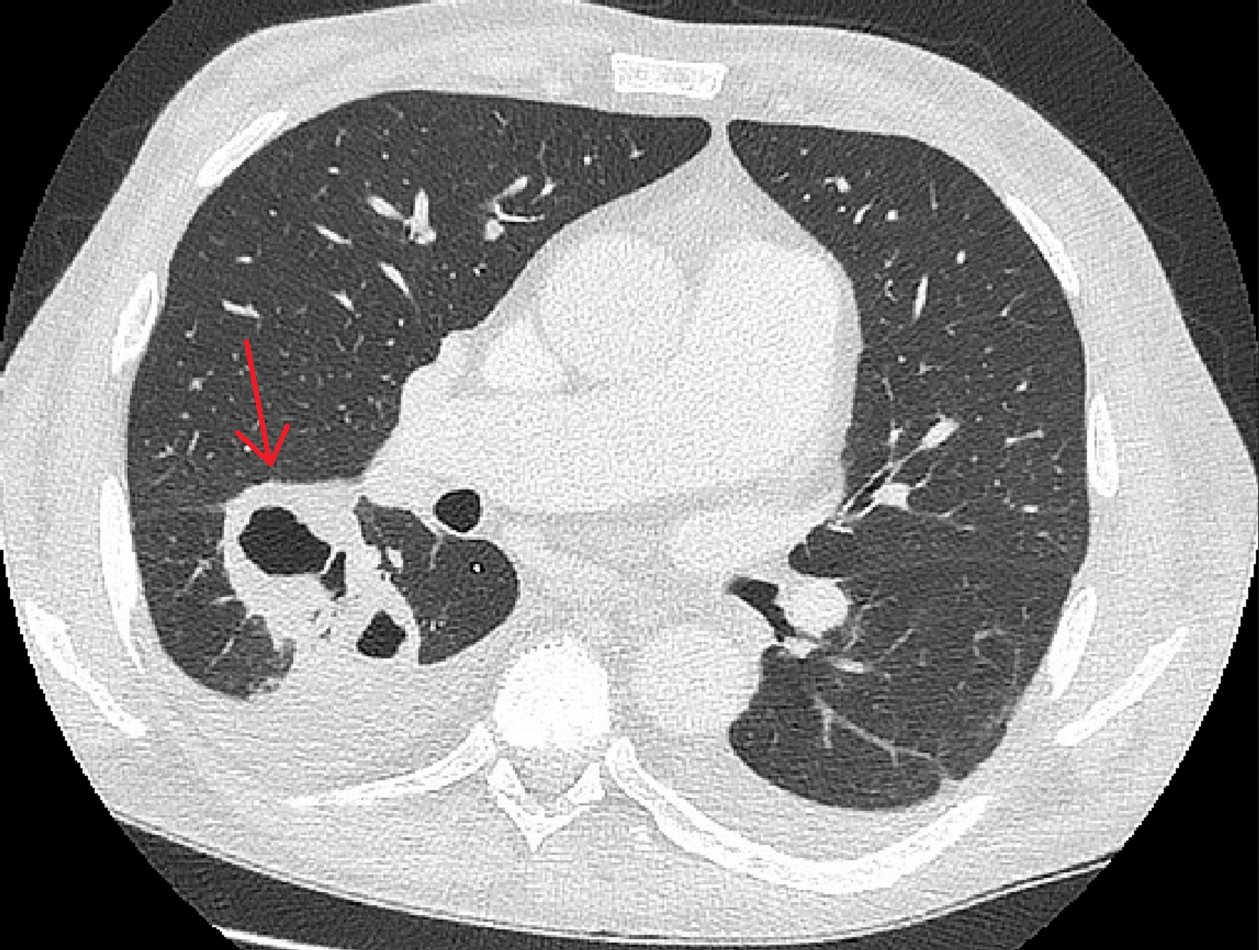
Chest computed tomography with visible lesions in segment VI of the right lung (red arrow) at the initiation of the treatment (July 2024).

On admission, he presented an enlarged, remodelled AVF on the left arm with dilated superficial veins on the left side of the chest. He also had peripheral oedema, most visible on his lower legs. His N-terminal pro-B-type natriuretic peptide (NT-proBNP) concentration was 13,593 pg/mL. Based on clinical symptoms (fatigue and non-specific generalised body pains) and repeated blood culture results, sepsis caused by *Staphylococcus haemolyticus* was diagnosed, and therapy with meropenem and vancomycin was initiated, according to the antibiogram. However, the radiological presentation of pneumonia strongly suggested mycosis. Both X-ray (Figure 1) and CT (Figure 2) revealed a few thick-walled oval structures located in the lower segments of the right lung. The largest lesion measured 98 x 62 x 65 mm and was connected to the segmental bronchi. Additionally, parenchymal opacities of the right lung were described. Based on these findings, invasive pulmonary aspergillosis (IPA) was suspected. Therefore, the patient was treated with antifungal medication - voriconazole. Due to cardiac and respiratory failure, he was temporarily disqualified from surgical intervention (lower bilobectomy). Furthermore, immunosuppressive therapy was adjusted by suspending mycophenolate mofetil. After 30 days of treatment, the decision to discontinue antibiotherapy was made based on improvement of the general status and normalisation of the inflammatory markers. After three days, meropenem was reintroduced due to a rapid increase in inflammatory markers.

**Figure 3:**
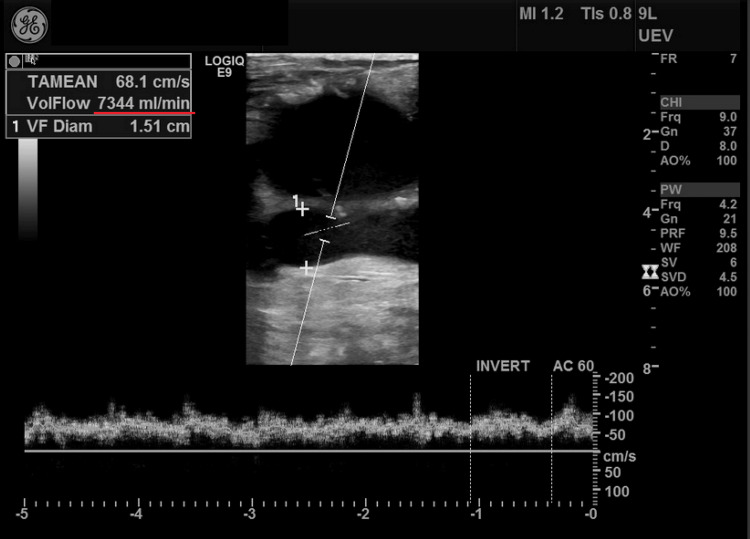
High-flow arteriovenous fistula in ultrasonography (flow rate underlined).

The patient required repeated HD for one month using an acute catheter. After the infection had been controlled, hypervolemia secondary to HF was managed with ultrafiltration. Following two weeks of treatment, the patient lost 15 kg, with successful reduction of peripheral oedema and improvement of general status. Due to an excessively high flow through the fistula (over 7000 mL/min) (Figure 3) and dilation of the venous part of the fistula (5 cm) along with an old thrombus (3.7 cm), the decision was made to remove the fistula to reduce cardiac insufficiency. The influence of AVF on cardiac insufficiency was based on right ventricle overload, visible in echocardiography (right ventricular systolic pressure (RVSP) 60 mmHg, pulmonary artery diameter 27 mm, tricuspid regurgitation pressure gradient (TRPG) 45 mmHg). At the end of August 2024, the fistula was excised with primary closure of the brachial artery and removal of the remodelled cephalic vein up to the axillary fossa. Prior to removal, the fistula was clamped for five minutes while heart function was monitored.

**Figure 4:**
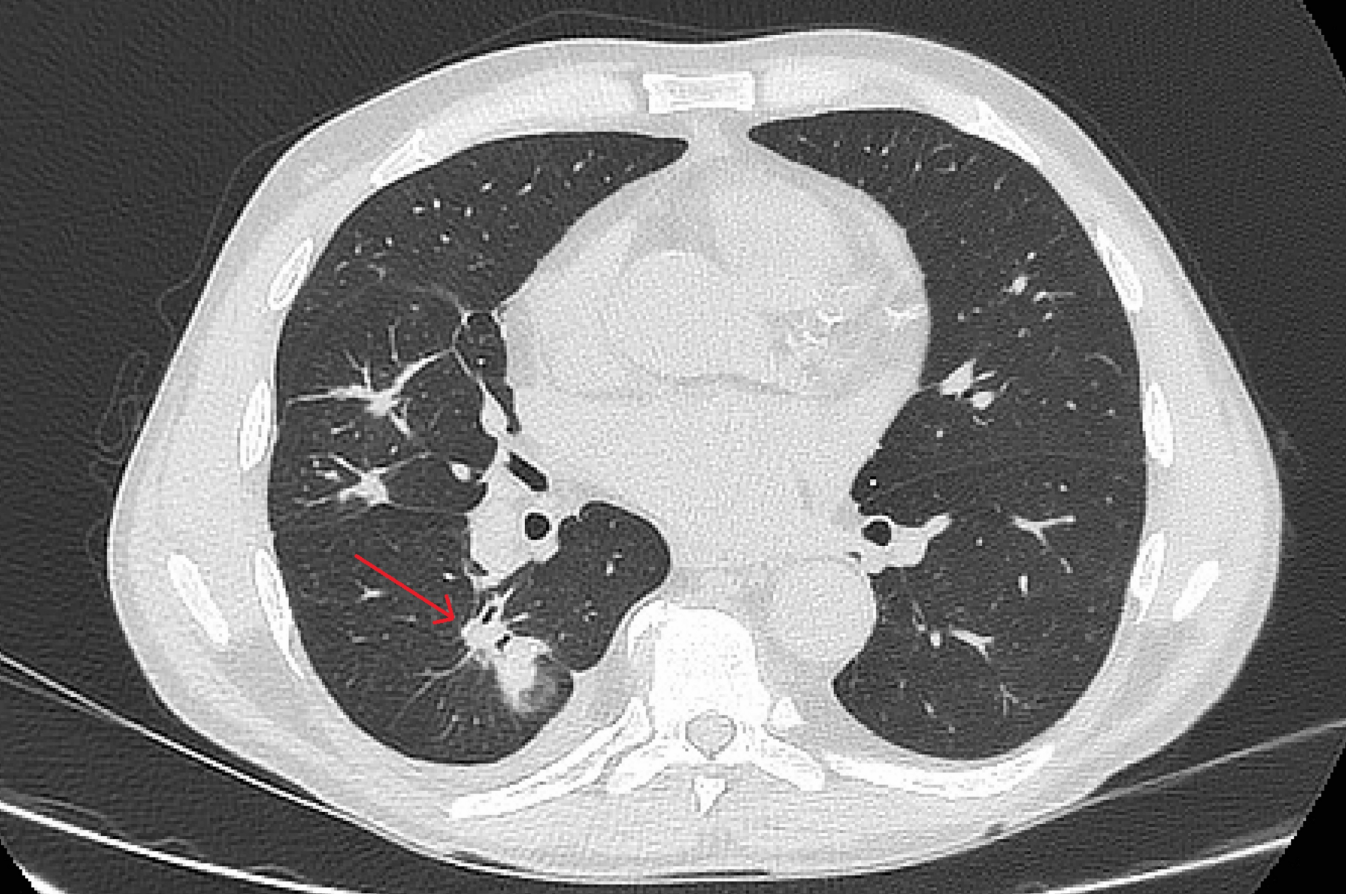
Follow-up chest computed tomography in March 2025 (red arrow indicating residual lung lesion in segment VI of the right lung).

After the surgery, *S. haemolyticus* bacteriemia recurred, so meropenem (for a total of 60 days) and vancomycin (for a total of 53 days) were continued. A subfascial haematoma (115 x 40 x 230 mm) was partially drained, which reduced the oedema of the upper limb. On the control chest CT scan, a reduction of lung lesions is visible (Figure 4), but it remained unsatisfactory. A decrease in pleural and pericardial fluid was also observed. After discharge at the end of September 2024, the patient continued antifungal therapy with voriconazole until December 2024 and stopped it on his own.

**Figure 5:**
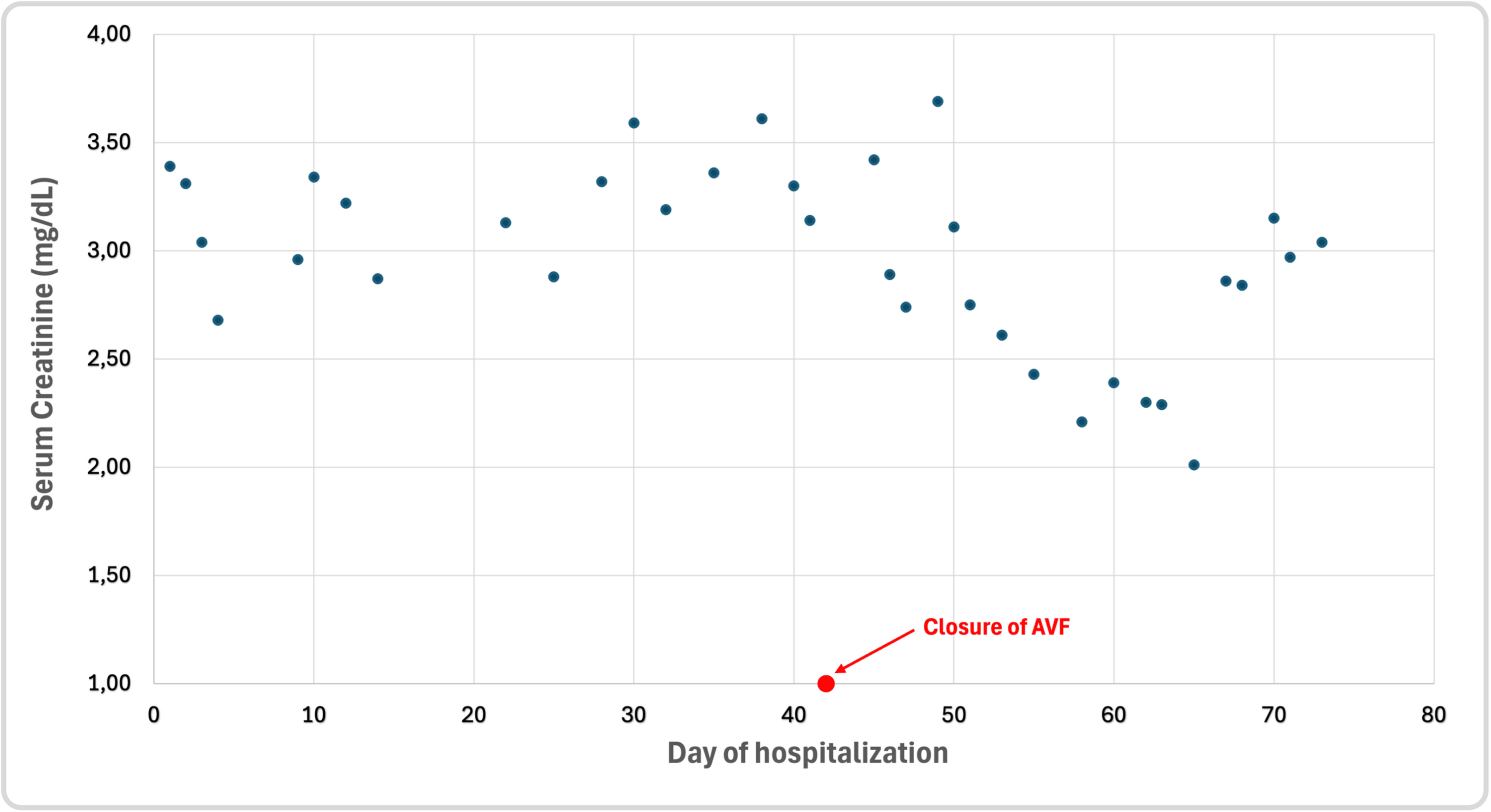
Serum creatinine concentrations during the hospitalisation period with the moment of arteriovenous fistul closure marked on x-axis.

The patient, after removal of the high-flow fistula, has not required HD, and SCr has returned to values between 2.01 and 3.15 mg/dL (eGFR from 35 to 21 mL/min/1.73 m²). Figure 5 visualises a decrease in SCr after the closure of the AVF, which may suggest a correlation between high-flow AVF and deterioration of graft function. However, the patient also required multiple interventions -- HD, followed by ultrafiltration and treatment of infection, which also likely contributed to the improvement of graft function. In the follow-up echocardiogram, cardiac status improved (RVSP decreased to 37 mmHg, TRPG decreased to 32 mmHg, and the diameters of the ventricles, atria, aorta, and inferior vena cava decreased). His NT-proBNP concentration dropped to 12,082 pg/mL.

The patient remains under the care of our transplant outpatient clinic. In the next control chest CT (last one in June 2025), further regression of the lung lesions was visible; most of them were described as fibrous. During the last outpatient visit (in January 2026) due to decrease in graft function (SCr 5.13 mg/dL, eGFR 12 mL/min/1.73 m²), the patient was referred to vascular surgeon to create a new AVF.

## Discussion

The impact of the high-flow fistula on cardiac function is clear. It causes dilation of all chambers, left ventricular hypertrophy, and an increase in left ventricular end-diastolic volume, leading to a clinical presentation of HF or pulmonary hypertension [8]. Progression of these changes reduces cardiac output, leading to hypoperfusion of peripheral organs, including the kidney graft. However, there is no clear-cut-off value for AVF flow that must be ligated. Typically, flows greater than 1500-2000 mL/min are considered harmful to the heart, but the threshold causing symptoms varies among patients. Prophylactic ligation of such AVF can prevent high-output HF [7]. According to Hetz et al., such procedures in asymptomatic patients lowered NT-proBNP concentrations and improved echocardiographic findings but did not affect SCr [6]. A meta-analysis by Zheng et al. confirmed that closure reduces ventricular mass index and left ventricular end-diastolic diameter, also reporting improved kidney graft function after ligation [9]. Another, more recent meta-analysis by Yasir et al. from 2024 demonstrated that ligation of symptomatic AVF is a safe and effective procedure. Furthermore, they proposed an AVF flow-to-cardiac output ratio exceeding 0.3 as a predictive marker of acute HF [10].

The functioning AVF also affects kidney graft function. According to Weekers et al., ligation of a functioning AVF in KTRs may accelerate a decline of kidney graft function [5]. On the other hand, Vajdic et al. found that patients with a functioning AVF had worse kidney graft function than those with a non-functional AVF [11]. However, most patients experienced spontaneous closure of the AVF, so it remains unclear whether this is a cause or a consequence of kidney graft function. As shown above, the results of the two studies are contradictory. Therefore, a clinical trial randomising patients to either close or leave the AVF open after transplantation could help clarify its impact on the kidney graft function.

The determinant of AVF management, as mentioned above, seems to be the flow, which is why, in some cases, banding procedures that restrict the venous part may sometimes be considered [12,13]. However, the potential complications of these procedures, such as reoperation, thrombosis, and infection, make ligation of the AVF the safest option. Sometimes, to prevent the development of thrombosis followed by thrombophlebitis, aneurysmal segments are excised [7].

A functioning AVF may serve as a good backup in the event of allograft failure. In such cases, it is important to note that graft function can be restored even after prolonged dialysis therapy [14], making the patent AVF potentially the most convenient and safe option for HD, especially for patients with other comorbidities such as infections. However, the patency of AVF was 82% after one year and 61% after five years in a centre that was diligent about monitoring vascular access [15], and it is likely lower in centres without an established protocol. According to Chan et al., in the United States, 65% of patients with kidney graft failure started HD with a catheter, and only 28% of patients used AVF [16]. The authors highlight the importance of earlier referral for access placement in patients at risk of graft failure. 

Diagnosis and classification of cardiorenal syndrome among KTRs might be challenging, as these patients often have a long history of chronic kidney disease. They might also have preexisting heart conditions. Both organs can be affected simultaneously by diabetes, hypertension, atherosclerosis, anaemia, or chronic inflammation and endothelial cell dysfunction [17]. Therefore, determining the primary dysfunction may be difficult, which is why it is consistent with the definition of secondary cardiorenal syndrome (type 5) [3].

No international guidelines cover post-transplant AVF management; only one position paper by the Vascular Access Society and the European Kidney Transplant Association addresses whether the AVF should be ligated after transplantation and, if so, when. The authors also emphasise the lack of solid data about the effects of AVF on kidney graft function and the cardiovascular system. They propose Doppler ultrasonography, combined with clinical examination, to assess AVF two months after transplantation. If the flow exceeds 1 L/min, echocardiography should be performed to establish a baseline. The next evaluation should be conducted one year after transplantation, and ligation, observation, or banding procedures should be considered. Decisions should be made by a multidisciplinary team consisting of a nephrologist, cardiologist, and vascular surgeon. The evaluation should be repeated every two years [7].

IPA is one of the most frequent fungal infections in solid organ transplant recipients, which might be lethal for them. Kidney function impairment requiring post-transplant dialysis, along with increased immunosuppressive therapy, might be a significant factor contributing to invasive aspergillosis [18]. The definitive diagnosis of IPA is challenging and involves multiple bronchoscopies with microbiological assessment. Therefore, most patients are treated before pathogen identification to improve their chances of survival [19]. The optimal treatment for IPA is controversial, but voriconazole remains the gold standard. It may interact with calcineurin inhibitors (tacrolimus and cyclosporine), so drug levels must be monitored carefully. To confirm the diagnosis, surgical resection with histological assessment is recommended [18]. 

Given the high mortality associated with IPA in KTRs, a well-considered clinical decision must be made regarding whether to discontinue immunosuppressive therapy, increasing the likelihood of successful pharmacological treatment at the cost of graft loss due to rejection.

## Conclusions

Closure of a high-flow AVF might contribute to the treatment of cardiac insufficiency, thereby improving glomerular filtration. Graft function might be restored, even after a prolonged course of HD therapy. Therefore, the decision whether to discontinue immunosuppressive therapy to treat the infection should be made carefully. AVF should be monitored regularly to prevent congestive HF.
